# The Complex Degradation Mechanism of Copper Electrodes on Lead Halide Perovskites

**DOI:** 10.1021/acsmaterialsau.1c00038

**Published:** 2022-02-02

**Authors:** Sebastian Svanström, Alberto García-Fernández, T. Jesper Jacobsson, Ieva Bidermane, Torsten Leitner, Tamara Sloboda, Gabriel J. Man, Gerrit Boschloo, Erik M. J. Johansson, Håkan Rensmo, Ute B. Cappel

**Affiliations:** † Condensed Matter Physics of Energy Materials, Division of X-ray Photon Science, Department of Physics and Astronomy, Uppsala University, Box 516, SE-751 20 Uppsala, Sweden; ‡ Division of Applied Physical Chemistry, Department of Chemistry, KTH - Royal Institute of Technology, SE-100 44 Stockholm, Sweden; § Young Investigator Group Hybrid Materials Formation and Scaling, Helmholtz-Zentrum Berlin für Materialen und Energie GmbH, Albert-Einstein Straße 15, 12489 Berlin, Germany; ∥ Uppsala-Berlin Joint Laboratory on Next Generation Photoelectron Spectroscopy, Albert-Einstein-Str. 15, 12489 Berlin, Germany; ⊥ Department of Chemistry, Uppsala University, Box 538, 75121 Uppsala, Sweden

**Keywords:** perovskite solar cell, X-ray photoelectron spectroscopy, stability, back contact, metal, interface chemistry

## Abstract

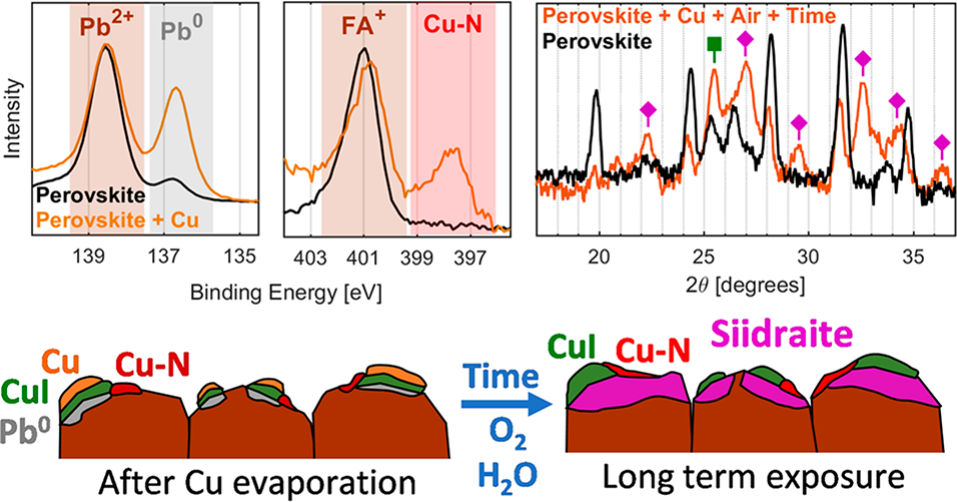

Lead halide perovskite solar cells have reached power conversion efficiencies during the past few years that rival those of crystalline silicon solar cells, and there is a concentrated effort to commercialize them. The use of gold electrodes, the current standard, is prohibitively costly for commercial application. Copper is a promising low-cost electrode material that has shown good stability in perovskite solar cells with selective contacts. Furthermore, it has the potential to be self-passivating through the formation of CuI, a copper salt which is also used as a hole selective material. Based on these opportunities, we investigated the interface reactions between lead halide perovskites and copper in this work. Specifically, copper was deposited on the perovskite surface, and the reactions were followed in detail using synchrotron-based and in-house photoelectron spectroscopy. The results show a rich interfacial chemistry with reactions starting upon deposition and, with the exposure to oxygen and moisture, progress over many weeks, resulting in significant degradation of both the copper and the perovskite. The degradation results not only in the formation of CuI, as expected, but also in the formation of two previously unreported degradation products. The hope is that a deeper understanding of these processes will aid in the design of corrosion-resistant copper-based electrodes.

## 1 Introduction

The rise of lead halide perovskite solar cells in the field of emerging solar cell technology has generated significant attention due to their high power conversion efficiency (PCE), now reaching over 25%,^1^ while being made of low-cost starting materials and using simple deposition techniques. However, commercialization of perovskite solar cells has been hindered by poor long-term stability. The main research effort for improving their stability has been focused on the perovskite absorber material, with the starting composition of MAPbI_3_ (where MA stands for methylammonium)^2^ evolving into more complex materials such as Cs_*x*_FA_*y*_MA_1–*x*–*y*_PbBr_*z*_I_3–*z*_ (where FA stands for formamidinium) with significant gains in stability as well as performance.^3^ Moreover, extensive research aimed at electron transport materials (ETM) and hole transport materials (HTM) has also been carried out, with additional efforts on the encapsulation of the solar cells for improving both stability and efficiency.^4,5^

However, the back-contact electrode and its effect on performance and stability has not been studied as extensively. One reason for this is that current research devices use gold back contacts, which, due to gold’s limited reactivity and suitable work function, allow for relatively stable devices and high efficiency.^6^ However, to what extent the Au layer is inert is a matter of discussion. This is exemplified by the work by Domanski et al., who found the migration of Au from the electrode both through the hole transport material and the perovskite layer to the front contact with a loss of efficiency as a result.^7^ Furthermore, gold electrodes are prohibitively expensive for use in commercial solar cells. Several inexpensive alternative electrode materials have been proposed, for example, carbon-based electrodes, conductive oxides, and cheaper metals (Ni, Al, W, Cu, Mo).^6,8^ Many of the metals require additional interlayers to protect them from oxidation; for example, a thin MoO_*x*_ interlayer has successfully been applied to protect Al electrodes from oxidation.^9^ However, without any additional interlayers, copper (Cu) is one of the most successful alternative metals, with good ductility and conductivity.^10^

There have also been several reports using copper electrodes on lead halide perovskites. Zhao et al. studied the reaction of copper in direct contact with MAPbI_3_ both in air and in a nitrogen atmosphere.^11^ They found that the copper contacts were stable in a nitrogen atmosphere, even under thermal annealing. However, when exposed to air, both the copper electrode and the perovskite showed signs of rapid degradation. Similarly, Ding et al. studied the Cu/MAPbI_3_ interface without exposure to oxygen.^12^ They found that the evaporation of copper did not cause significant degradation of the perovskite surface or the copper electrode. Inverted perovskite solar cell devices using copper electrodes with C_60_ as barrier layers show good stability and achieve efficiencies above 20%.^11,13−15^ Deng et al. also studied copper electrodes on devices using both MAPbI_3_ and FA_0.4_MA_0.6_PbI_3_ and C_60_ barrier layers. While both were stable for 20 days, the efficiency of the FA_0.4_MA_0.6_PbI_3_ had decreased significantly at day 30,^16^ suggesting some contribution of the organic FA cation. Finally, Udalova et al. showed that copper could react with MAI, resulting in the formation of MACu_2_I_3_ and CuI.^17^

Additionally, copper-containing hole transport materials have been used successfully in conjunction with lead halide perovskites, which indicates compatibility. The highest efficiencies for cells with Cu-containing HTL (above 20%) have been reached with CuSCN without any significant unresolved stability issues.^18−20^ Even more encouraging is the use of CuI as a HTM with efficiencies up to 16.8% and excellent stability.^21−23^ CuI is the expected product in the reaction between copper and iodine-containing materials, which should allow copper electrodes to be chemically self-passivating in contact with the perovskite. This reaction can be induced intentionally, as demonstrated by Nazari et al.,^24^ or occur as a result of incomplete coverage of the selective layers. The passivation can also be carried out before device assembly. Metallic copper foils can be treated to form a surface layer of CuI before perovskite deposition, resulting in flexible substrate and hole selective contact.^25^ Similarly, copper nanowires can also be treated to form a surface layer of CuI, resulting in a charge carrier selective material that maintains the high conductivity of the Cu metal.^26^

In addition to interface design, a number of studies have shown that inclusion of a small amount of Cu ions into the perovskite layer can increase both the efficiency and stability of perovskite solar cells.^27−29^ The ions improve crystallinity and phase stability and make the perovskite more n-type by shifting the Fermi level toward the conduction band.^27,29,30^ There is some uncertainty whether the Cu^+^ ions occupy the Pb^2+^ site^30,31^ or the site of the monovalent cation.^29^ Ming et al. calculated the formation energy of Cu^+^ interstitials in MAPbI_3_ to between −1.1 and +0.4 eV depending on if the material is p- or n-type.^31^ Furthermore, the diffusion barrier of Cu^+^ ions was determined to be 0.42 eV, which is comparable to that for the highly mobile I^–^. Although copper easily forms interstitials and migrates through the perovskite, the defects induced by Cu^+^ are generally benign and therefore should not affect charge carrier transport significantly.^31^

Most studies discussed above indicate that copper is a suitable electrode material for commercial lead halides perovskite devices given its low cost and potential of increasing stability through self-passivation. The latter could help prevent long-term stability issues induced by direct contact between copper and perovskite, which might be created by incomplete coverage of selective contacts. This could be intentional (for example, scribing of modules), accidental damage or pin holes, the probability of which becomes increasingly more likely as the area of the cells are scaled up.^32^ Additionally, there have been studies proposing a one-step deposition of Cu/CuI back contact, with the CuI forming from the direct contact between the perovskite and copper.^33^ However, the chemical reactions that occur in the interface between the perovskite and copper are not well-known, especially for other perovskite compositions than MAPbI_3_. A direct investigation of these reactions is therefore of interest in addition to studies investigating the stability of complete devices. In the present study, we have therefore investigated the interaction between thermally evaporated copper and the perovskite surface with the aim of determining how the two materials interact and which pathways could contribute to their degradation. This study is therefore intended to provide a fundamental understanding of the reactions, which can occur between lead halide perovskite and copper rather than to study the degradation of a specific solar cell design.

The main perovskite composition used in this study was Cs_0.17_FA_0.83_PbI_3_, representing a stable lead halide perovskite that offers several advantages. First, it is stable in the black phase (which is not the case for pure FAPbI_3_),^34^ thereby representing a viable solar cell composition. Second, the composition has a single halide, thereby avoiding effects such as differential halide reactivity or halide phase separation.^35,36^ It contains only FA^+^ as an organic A-site cation, therefore limiting the degradation mechanism to a single organic cation. Comparison measurements were also carried out on MAPbI_3_, one of the most widely studied lead halide perovskite, as well as PbI_2_ and FAI (precursor salts for Cs_0.17_FA_0.83_PbI_3_). Detecting electronic and chemical changes in the thin interface between the perovskite and copper requires a technique with suitable surface sensitivity as well as elemental and chemical sensitivity. Photoelectron spectroscopy (PES) fulfils such a requirement and allows for both ex situ and in situ investigation of evaporated thin Cu films. Both in-house and synchrotron-based PES were the prime tools used in the present investigation. These measurements were complemented by X-ray diffraction (XRD) to study structural changes in the bulk of both the deposited copper films and the perovskite substrates.

## 2 Experimental Methods

### 2.1 Sample Fabrication

The perovskites and lead halide thin film samples were deposited on FTO/TiO_2_ substrates prepared in the following way: The FTO glass was cleaned in an ultrasound bath in three 30 min steps with RBS 50 detergent, ethanol, and finally acetone. The substrates were subsequently treated in a UV-ozone cleaner for 10 min. An electron transport layer of TiO_2_ was deposited on the cleaned FTO substrates using spray pyrolysis. The spray solution consisted of ethanol, acetyl acetone, and titanium diisopropoxide (30% in isopropyl alcohol) in the proportions of 90:4:6 by volume with air at a base pressure of 1 bar as a carrier gas. The FTO substrates were heated to 450 °C on a hot plate and kept at that temperature for 15 min prior to the spraying. Ten milliliters of spray solution was used to cover 200 cm^2^ of substrates, giving a compact layer of anatase with a thickness of around 20–30 nm. On top of the compact layer, a mesoporous scaffold of TiO_2_ nanoparticles was deposited by spin-coating. TiO_2_ paste (30 NR-D) and was dissolved in ethanol at a concentration of 150 mg/mL. On each substrate (1.5 × 2.5 cm), 50 μL of the TiO_2_ solution was applied and spin-coated at 4000 rpm, with an acceleration of 2000 rpm/s for 10 s. Both the compact and mesoporous TiO_2_ layers were sintered at 450 °C in air on a hot plate/oven for 30 min after deposition and then slowly cooled to ambient temperature.

Perovskite precursor solutions were prepared in a glovebox with an argon atmosphere. Stock solutions were prepared in advance, whereas the final precursor solution was prepared just before perovskite deposition. Anhydrous DMF/DMSO in the proportion of 4:1 was used as solvent. To ensure that all of the precursors were completely dissolved, the solutions were heated under stirring on a hot plate at 100 °C for 20 min and then cooled to room temperature just before use. For the perovskite with the composition Cs_0.17_FA_0.83_PbI_3_, two master solutions were prepared: (a) 0.9 mol PbI_2_ and 0.9 mol FAI per liter of solvent and (b) 0.9 mol PbI_2_ and 0.9 mol CsI per liter of solvent, which were mixed in a the proportion of *a*/*b* = 83:17. In the case of MAPbI_3_, a perovskite precursor solution of 1.25 mol PbI_2_ and 1.14 mol MAI per liter of solvent was prepared. The solution therefore contains an excess of PbI_2_, which improves the quality of the films.^37^ However, XRD shows that no PbI_2_ phase is present (see below).

The perovskite precursor solutions were spin-coated in a glovebox with an inert atmosphere. First, 75 μL of the precursor solution was spread over the substrate (1.5 × 2.5 cm), which thereafter was spin-coated using a two-step program. The first step was a spreading step using a rotation speed of 500 rpm with an acceleration of 500 rpm/s for 5 s. That step was immediately (without pause) followed by the second step, where the films were spun at 4500 rpm for 30 s using an acceleration of 2225 rpm/s. During the second step, when approximately 15 s of the program remained, 200 μL of anhydrous chlorobenzene was dropped on the spinning film with a hand-held automatic pipet. This last step, known as the antisolvent method, has a large impact on film morphology. Directly after spin-coating, the films were placed on a hot plate at 100 °C, where they were annealed for 30–60 min. The PbI_2_ thin film samples and FAI thin film samples were prepared by spin-coating 0.8 M solutions of the corresponding material in DMF using a one-step program and a rotation speed of 3500 rpm with an acceleration of 3500 rpm/s for 20 s. This was followed by 30 min of annealing at 70 °C. Samples were stored and transported in a dark and low-moisture atmosphere before evaporation and between measurements.

### 2.2 Measurements

The photoelectron spectroscopy measurements were carried out at the CoESCA endstation at the UE-52 PGM beamline at the BESSY II electron storage ring operated by the Helmholtz-Zentrum Berlin für Materialien und Energie. The unique feature of the CoESCA endstation is the presence of two angle-resolved time-of-flight (ArTOF) spectrometers, one ArTOF-10k and one ArTOF2-EW, allowing two concurrent measurements of the same sample.^38^ The high transmission of the ArTOF spectrometers allows the X-ray flux to be low enough to avoid any beam damage to the perovskites.^39^ The X-ray beam and two spectrometers used in the setup are located in one plane, and the spectrometers are at an angle of 57° on either side of the X-ray beam. The single bunch X-rays were generated with pulse picking by resonant excitation (PPRE)^40^ using the UE-52 undulator and monochromated using a plane grating monochromator beamline. The measurements were carried out at two different photon energies, 535 and 1060 eV. Additionally, a photon energy of 758 eV was used for calibrating the energy scale of the spectrometers by setting the binding energy difference of I 4d and I 3d to 569.9 eV.

To clean the surface, the perovskite samples were sputtered with argon at a pressure of 5 × 10^–6^ mbar for 60 min with an acceleration voltage of 350 V and emission current of 10 mA. The samples were kept at a grazing angle relative to the normal of the ion gun. After being sputtered, the sample was annealed by heating to 90 °C for 30 min before being allowed to cool again. To ensure that the reactions observed were not due to the sputtering, a separate sample was not sputtered. For evaporation of copper, a custom-made evaporator with a high resistance, alumina-coated tungsten coil was used. This allowed the use of a smaller power supply and prevented tungsten contamination. Before evaporation, the deposition chamber was pumped down to below 10^–9^ mbar, and the evaporation source was degassed by heating it to just below the evaporation temperature to avoid exposure to oxygen. During the evaporation, the temperature was increased until boiling could be observed, and the evaporation was allowed to continue for 10 s before the temperature was decreased. After evaporation, the samples were transferred into the measurement chamber without breaking the vacuum within 10 min after evaporation. To expose the samples to air, they were placed in a chamber which was ventilated with air for 10 min before being pumped down to vacuum again. Any longer exposure was done by simply removing the samples from the vacuum chamber and storing them in air.

Measurements of PbI_2_ and FAI were carried out at the SPECIES beamline at MAX IV. The X-rays at the SPECIES beamline were generated using the EPU61 undulator (5th harmonic) with an aperture slit of 1 × 1 mm with an exit slit of 40 μm. This gives an estimated intensity of about 4 × 10^11^ photons/s, with an elliptical spot size of 100 × 30 μm, resulting in an estimated X-ray flux density of 4 × 10^15^ photons/s/cm^2^. The photoelectrons were detected using a SPECS Phoibos 150 NAP with a pass energy of 100 eV and a step size of 0.1 eV.^41^ All measurements shown were carried out at pressures below 10^–9^ mbar in the analysis chamber. Evaporation was carried out using the same setup as the CoESCA endstation, except that no sputtering was carried out. Exposure to oxygen was carried out in the ambient pressure manipulator, whereas exposure to air was carried out by ventilating the load lock, similar to that at the CoESCA endstation.

For the in-house measurements, the copper electrodes were evaporated directly on the perovskite using a Leica EM MED020 thermal evaporator. The evaporation was carried out at a pressure below 7 × 10^–3^ mbar, and the thickness was measured using a Leica EM QSG100 quartz microbalance. Half of the film was masked to serve as a reference.

In-house X-ray photoelectron spectroscopy measurements were performed on a Physical Electronics Quantera II using Al Kα (1486.6 eV) X-rays at a pressure below 7 × 10^–9^ Torr. The survey spectra were recorded using a pass energy of 280 eV with an energy step of 1 eV, and all other measurements were carried out using a pass energy of 55 eV and an energy step of 0.1 eV.

The photoelectron spectra of the core levels were fitted using a pseudo-Voigt function^42^ with a polynomial, Herrera-Gomez,^43^ and Shirley background.^44^ To estimate the ratios between elements, the area of the photoelectron peaks was normalized to the photoionization cross section.^45^

XRD was measured at ambient conditions using a Siemens D5000 Th-2Th X-ray diffractometer using Cu Kα (λ = 1.5406 Å). The incident angle was 2°, the step size was 0.02°, and the dwell time was 500 ms.

## 3 Results and Discussion

### 3.1 Cs_0.17_FA_0.83_PbI_3_ before Evaporation of Copper

**Figure 1:**
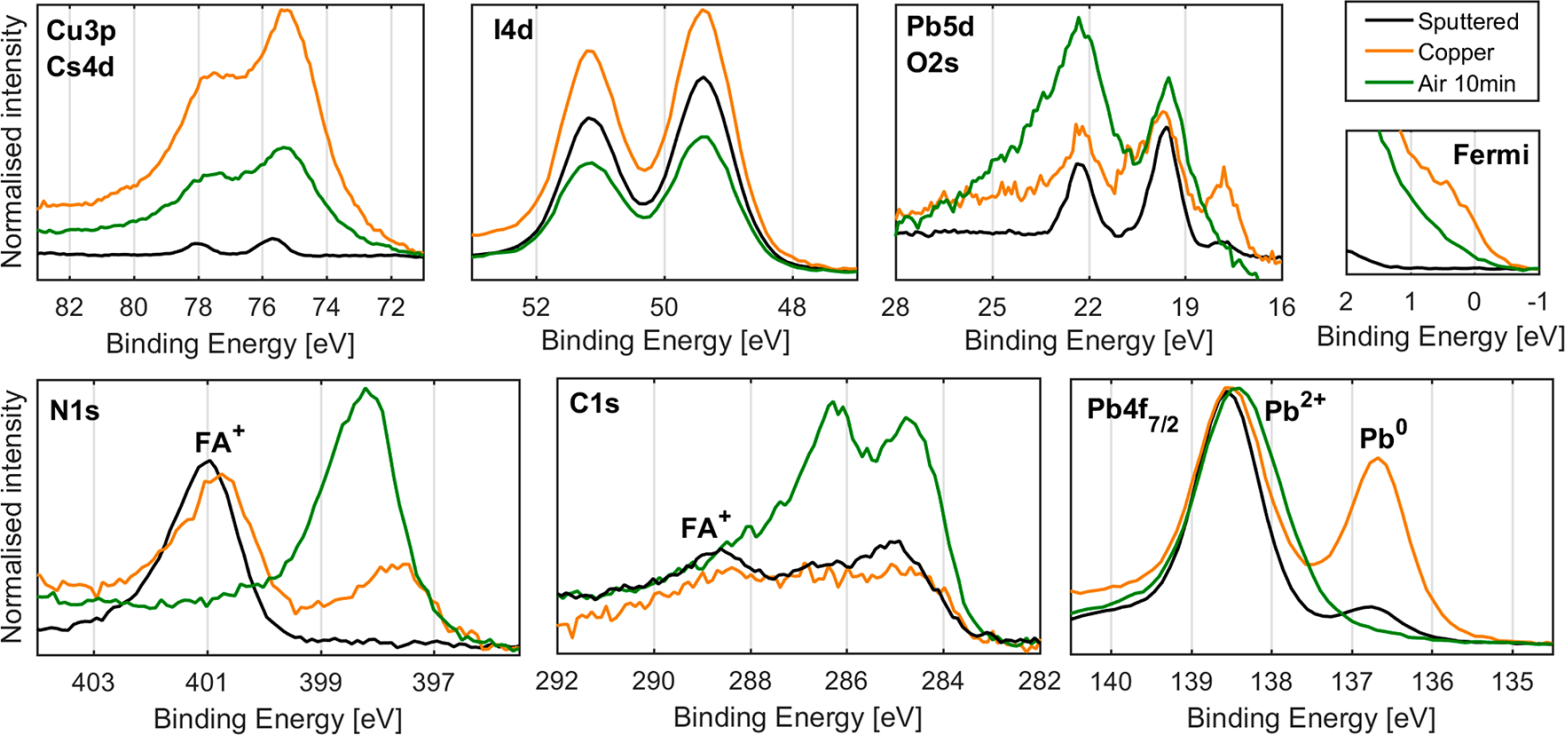
Cu 3p, Cs 4d, I 4d, Pb 5d, O 2s, C 1s, N 1s, and Pb 4f_7/2_ core levels and the Fermi level of the Cs_0.17_FA_0.83_PbI_3_, normalized to the height of the Pb^2+^ component from Pb 4f. The N 1s and C 1s core levels were measured using ArTOF-10k and the remaining core levels using the ArTOF2-EW with a photon energy of 535 eV. The binding energy was calibrated by setting the binding energy of the I 4d core level to 49.4 eV.

In situ measurements of the interface between evaporated copper and thin films of Cs_0.17_FA_0.83_PbI_3_ were carried out at the CoESCA endstation at the BESSY II synchrotron. The endstation allows rapid transfer between sample preparation and treatment to measurement without breaking vacuum (pressure <10^–8^ mbar). Before evaporation of copper, the surface of the Cs_0.17_FA_0.83_PbI_3_ was investigated with photoelectron spectroscopy. Measurements were carried out with a photon energy of 535 eV, resulting in a probing depth of 1.9 nm for N 1s, 2.6 nm for C 1s, 3.7 nm for Pb 4f, and 4.1–4.5 nm for Cu 3p, Cs 4d, I 4d, and the valence band.^46^ Due to this surface sensitivity, even a thin layer of surface contaminants will significantly affect the measurements. For our samples, this resulted in a very weak N 1s signal from the FA^+^ cation but large O 2s and C 1s signals from the surface contamination. Therefore, before evaporating copper, the sample was sputtered and thermally annealed in vacuum to remove surface contaminants and expose a clean perovskite surface. Photoelectron spectra of Cs_0.17_FA_0.83_PbI_3_ before and after sputtering are shown in Figure S1 and indicate the presence of a perovskite material with very limited amounts of adventitious carbon and oxygen components. The removal of surface contaminants prevents any surface reactions between them and copper and allows us to focus on the perovskite/copper interface. Figure 1 shows the N 1s, Pb 4f_7/2_, I 4d, Cs 4d, and Cu 3p core levels and the Fermi edge after sputtering, copper evaporation, and exposure to air for 10 min. All spectra were normalized to the height of the Pb^2+^ component of the Pb 4f core level.

After sputtering and annealing, we observe the following core levels originating from the perovskite: a Pb 4f_7/2_ signal (138.5 eV) associated with Pb^2+^, a I 4d doublet signal (49.4 and 51.1 eV) associated with I^–^, a Cs 4d doublet signal (75.7 and 78.0 eV) associated with Cs^+^, and finally a N 1s signal (401.0 eV) and a C 1s signal (288.7 eV) associated with the FA^+^ cation. These binding energies fall within the range expected for a perovskite of this composition.^47^ However, we also observe a Pb 4f_7/2_ signal at lower binding energy (136.7 eV), associated with Pb^0^, representing about 10% of total Pb intensity and two minor C 1s signals (284.8 and 286.3 eV) attributed to remaining adventitious carbon. Taken all together, this indicates a relatively clean perovskite surface but with small amounts of metallic lead formed during sputtering.

### 3.2 Cs_0.17_FA_0.83_PbI_3_ after Evaporation of Copper

A thin layer of copper was evaporated on the surface and then immediately measured without exposure to atmosphere (Figure 1). After evaporation, we are able to detect the presence of the evaporated copper by a Cu 3p doublet at 75.2 and 77.7 eV in the same binding energy region as Cs 3d.

Although there are clear changes in the spectra, we are still able to detect some perovskite signals (Pb^2+^, I^–^, and FA^+^) at binding energies close to those expected from the pristine material, indicating thin or incomplete copper coverage. The exception is the Cs 4d core level which overlaps with Cu 3p and is therefore not visible after the deposition. There is a slight increase in the intensity of the I 4d doublet (associated with I^–^) relative to Pb^2+^, whereas the intensity of the N 1s signal (associated with FA^+^) remains relatively unchanged. Taken together, this indicates that some perovskite remains intact at the surface after copper deposition.

However, there are also significant material changes occurring upon copper deposition, which mainly are observed in the Pb 4f and N 1s spectra. There is a new lower binding energy N 1s signal at 397.6 eV contributing to about 34% of total N 1s intensity as well as a significant increase in the Pb 4f_7/2_ signal associated with Pb^0^ to about 41% of total Pb. Finally, the appearance of a Fermi edge indicates the presence of metallic states (Pb^0^ and Cu^0^).

The formation of Pb^0^ and a new nitrogen signal shows that the deposited copper induces the degradation of the perovskite surface. Both features also form in similar relative amounts (about 30–40% of total N or Pb), indicating that they might form from the same process. However, going from Pb^2+^ to Pb^0^ requires the reduction of lead and the corresponding oxidation of another component, most likely Cu^0^ to Cu(I). This likely results in the formation of CuI as there is an increase of the I^–^/Pb^2+^ ratio, indicating that some of the I^–^ bound to another species. No changes are observed during measurement (Figure S2), suggesting that these changes occur during evaporation.

### 3.3 Cs_0.17_FA_0.83_PbI_3_/Copper Interface after Exposure to Air

The sample discussed above was then exposed to air for 10 min before being transferred to a vacuum and measured again. After exposure to air, the Pb 4f_7/2_ signal attributed to Pb^0^ disappears, whereas the Pb 4f_7/2_ signal attributed to Pb^2+^ becomes significantly broader (from 1.0 to 1.2 eV full width at half maximum). This conversion from Pb^0^ to Pb^2+^ results in an increase in the latter, and due to the normalization, this results in a decrease in the Cu 3p and I 4d signal intensity relative to Pb^2+^. There is a significant decrease in the intensity of the Fermi edge, which suggests a decrease, but not complete disappearance, in the number of metallic states. As there is no Pb^0^ at the surface after oxygen exposure, these must be attributed to Cu^0^. Furthermore, a broad signal appears at about 23 eV, which can be assigned to O 2s and, which together with the changes in the Pb 4f signal, indicates the formation of Pb–O compounds.

There is a significant increase in the relative intensity of the new N 1s signal and a shift to about 398.2 eV. There is also a new C 1s signal at 284.6 eV, attributed to adventitious carbon, and a new C 1s signal at about 286.2 eV, which could be linked to the new nitrogen signal. Finally, the exposure to air also results in the complete disappearance of the N 1s signal attributed to the FA^+^, indicating the degradation of the remaining perovskite at the surface.

When the sample was exposed to air for 14 h, we observed higher intensities of the signals relating to the formation of Pb–O, the new nitrogen compounds and CuI, as well as a complete disappearance of metallic copper (Figure S3). However, there are no changes in the signals during measurements in vacuum, indicating that the reaction only occurs when exposed to air (Figure S2).

Separate measurement of the Cu 2p core level were performed using a photon energy of 1060 eV (Figure S4). In these measurements, it is not possible to distinguish Cu^0^ and Cu(I). However, after 37 h of exposure to air, the formation of Cu(II) was observed through a peak shift of Cu 2p and the appearance of satellite peaks, which are associated with Cu(II) compounds.^48^

### 3.4 Comparison to Other Perovskite Samples

To ensure that the effects observed were not an effect of the sputtering or annealing of the perovskite surface, we performed the experiment on a nonsputtered sample (Figure S5). In this case, we observe the formation of the same species as on the sputtered sample, however, with a smaller amount of metallic lead (8% of Pb) after copper evaporation. This suggests that the formation of Pb^0^ is impacted by the conditions of the surface during evaporation. As indicated above, without sputtering, only a small amount of perovskite (FA^+^) nitrogen could be observed with the surface sensitive measurement at 535 eV. However, a significant increase in nitrogen intensity is observed for the nonsputtered perovskite upon copper evaporation and subsequent exposure to air.

Furthermore, we studied ex situ samples of Cs_0.17_FA_0.83_PbI_3_ and MAPbI_3_ 24 h and 2 months after the evaporation of 40 nm of copper, that is, a significantly larger amount than in the in situ measurements discussed earlier (Figures S6 and S7). We were still able to detect a Pb 4f signal after evaporation, suggesting that the copper does not form a continuous covering layer similar to what was observed by Ding et al.^12^ The changes in the Cs_0.17_FA_0.83_PbI_3_ signals appear very similar to what we observed after exposure to air in the in situ measurements, that is, a complete degradation of the perovskite surface and the formation of new nitrogen species. The new nitrogen species were still observed 2 months after evaporation. Similarly, the MAPbI_3_ sample showed no MA^+^ signal after evaporation of copper, indicating a complete degradation of the perovskite surface. However, unlike Cs_0.17_FA_0.83_PbI_3_, no new nitrogen species were observed. Also, significantly more Cu(II) was observed for the Cs_0.17_FA_0.83_PbI_3_ sample than for the MAPbI_3_ sample, suggesting that the presence of FA^+^ accelerates the oxidation of copper.

### 3.5 Reactions of Copper with Perovskite Precursors

**Figure 2:**
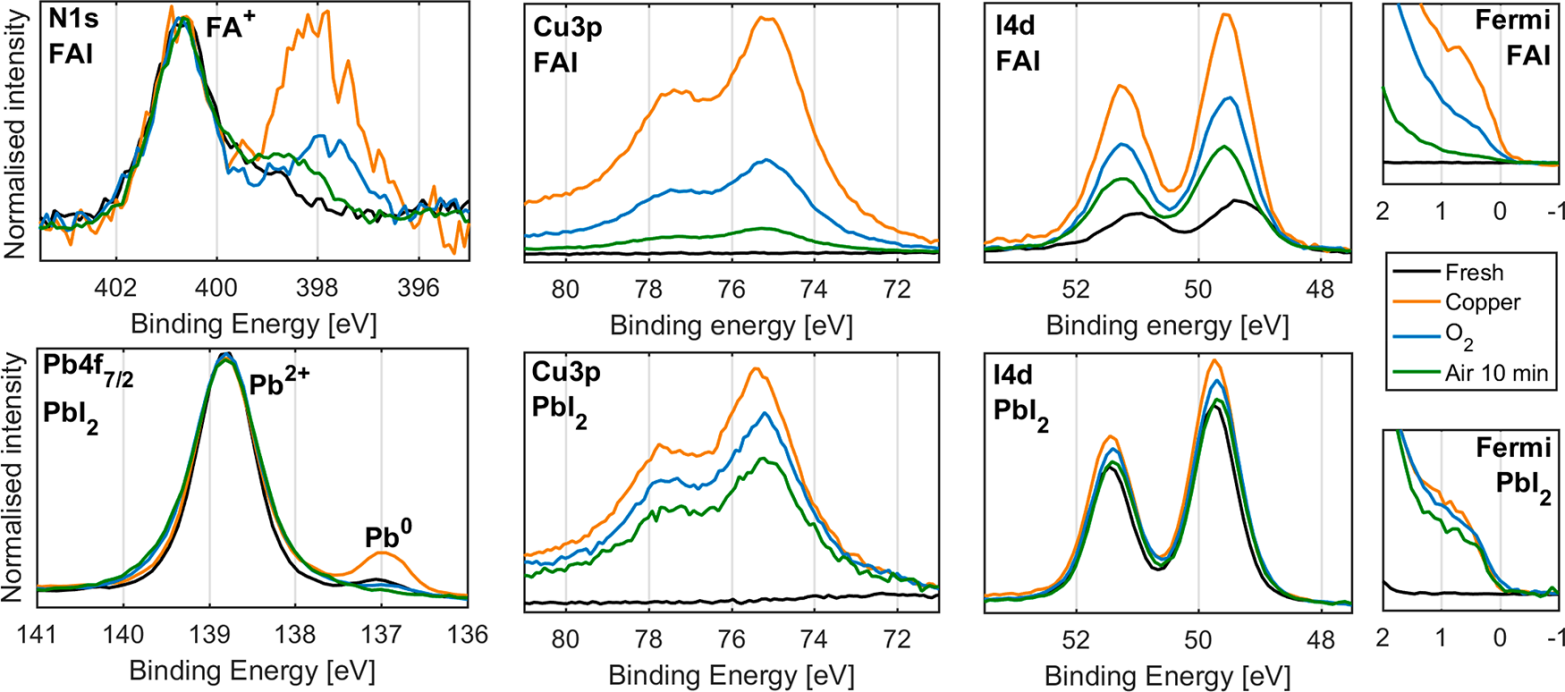
Top: The N 1s, Cu 3p, I 4d core level, and Fermi edge of the FAI sample. Measured at the SPECIES beamline using a photon energy of 535 eV, intensity normalized and energy calibrated against the N 1s (FA^+^) signal at 400.7 eV. Bottom: The Pb 4f, Cu 3p, I 4d core levels, and Fermi edge of the PbI_2_ sample. Measured at the SPECIES beamline using a photon energy of 535 eV, intensity normalized and energy calibrated against Pb 4f_7/2_ at 138.8 eV.

To determine if the reactions occur only with the perovskite or even separately with the lead iodide (PbI_2_) and the organic halide (FAI) precursors, we also investigated the interface of copper evaporated on thin films of these materials. Figure 2 shows the N 1s, Pb 4f, Cu 3p, I 4d, and Fermi edge of FAI (top) and PbI_2_ (bottom) thin films before and after evaporation of copper, after exposure to 0.5 mbar of oxygen for 40 min, and finally after exposure to air for 10 min.

For the FAI thin film, we observe an I 4d doublet at 49.3 and 51.0 eV, attributed to I^–^, and a N 1s signal at 400.7 eV, attributed to the FA^+^ cation before evaporation of copper. The latter also shows a lower binding energy shoulder; however, the cause of this shoulder is not known. After the evaporation of copper, we observe a new Cu 3p signal at 75.1 and 77.6 eV and the appearance of a Fermi edge, indicating the presence of metallic copper. There is a shift of the I 4d signal, attributed to I^–^, by about +0.3 eV, possibly due to the formation of CuI. Furthermore, there is a significant increase in the I 4d intensity relative to FA^+^ and the formation of a new N 1s signal at 398.0 eV, contributing to about 50% of the total N 1s intensity. The sample was then exposed to O_2_ at a pressure of 0.5 mbar for 40 min, resulting in a significant decrease in intensity of the Cu 3p signal, Fermi edge, and the new nitrogen signal (decreasing to 33% of total N), relative to FA^+^. This was followed by exposure to air for 10 min, resulting in a further decrease in intensity of the Cu 3p signal and the Fermi edge but no change in the intensity of the new nitrogen species, relative to FA^+^. The new nitrogen species also shifted to about 398.9 eV, similar to what we observed for Cs_0.17_FA_0.83_PbI_3_.

Turning to the PbI_2_ sample, we observe a single Pb 4f_7/2_ signal at 138.8 eV, attributed to Pb^2+^, and a single I 4d doublet at 49.7 and 51.4 eV, attributed to I^–^ before evaporation. There is also a small amount of Pb^0^, as indicated by the small Pb 4f_7/2_ signal at 136.9 eV. After evaporation of copper, we observe the appearance of a Cu 3p signal at 75.2 and 77.7 eV and a significant increase in the intensity of the Pb^0^ signal. There is also the appearance of a Fermi edge, indicating the presence of metallic states (Cu^0^ and Pb^0^). After exposure to O_2_ at a pressure of 0.5 mbar for 40 min, we observe a significant decrease in the signal attributed to Pb^0^ and a slight decrease in Cu 3p signal relative to Pb^2+^. This trend continues after exposure to air for 10 min with the disappearance of the Pb^0^ signal and a further decrease in Cu 3p signal relative to Pb^2+^. However, after both steps, there is only a slight decrease in the intensity of the Fermi edge, showing that most metallic states still remain and therefore stem from Cu^0^.

**Figure 3:**
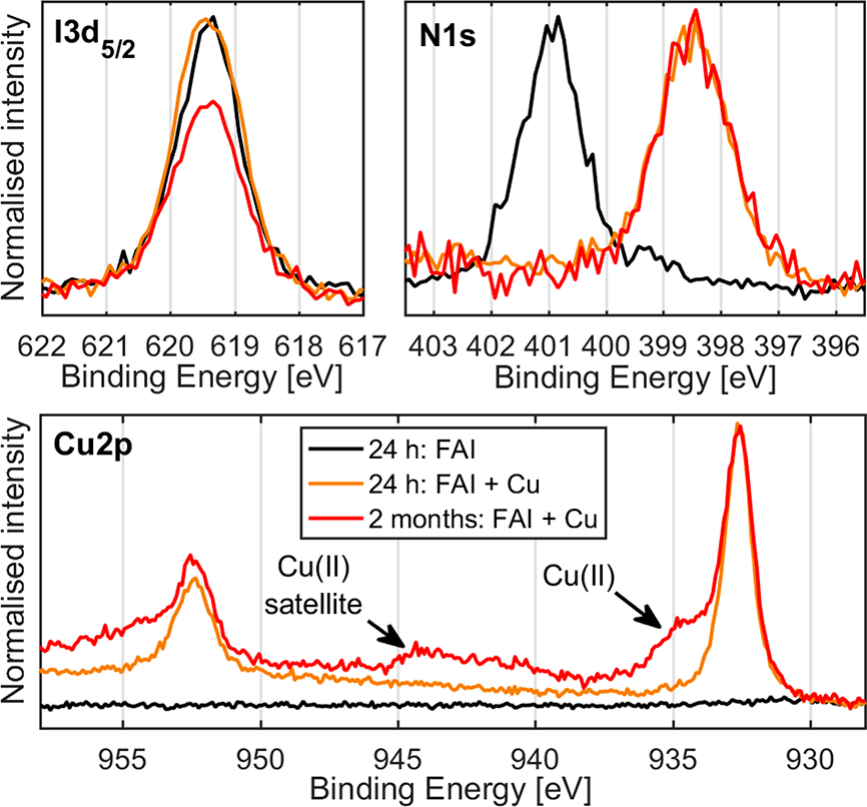
I 3d_5/2_, N 1s, and Cu 2p core levels of the FAI sample 24 h and 2 months after evaporation of copper. Measured using Al Kα, intensity normalized against total N 1s intensity, and energy calibrated against I 3d_5/2_ at 619.4 eV.

Ex situ measurements performed 24 h as well as 2 months after evaporation of 40 nm of copper on a thin film of FAI are shown in Figure 3. Within 24 h after evaporation, we observe that there is a very small change in the relative intensity of I 3d_5/2_ to N 1s between the region with and without copper. This indicates that the iodide to nitrogen ratio remains constant at around 1 I per 2 N both before and after evaporation. However, after 2 months, there is a significant decrease in the intensity of the I 3d signal relative to N 1s, indicating that the I/N ratio has decreased. There is no Fermi edge visible (Figure S8), indicating that no Cu^0^ remains at the sample surface. In the Cu 2p signal, we observe no Cu(II) within 24 h of evaporating but instead a signal consistent with Cu(I), whereas 2 months after evaporation, we observe the formation of Cu(II) corresponding to about 43% of total Cu. However, most notable is that there is no change in the Cu(I) ratio relative to N 1s intensity, strongly suggesting that these are a part of the same compound. The cross section normalized intensities give a Cu(I)/N ratio of 1.3, that is, slightly above 1.

### 3.6 Interfacial Reactions of Perovskite and Perovskite Precursors with Copper

**Figure 4:**
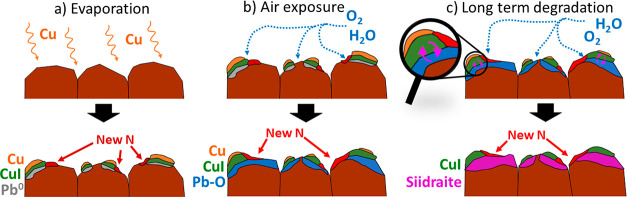
Schematic illustration showing the different treatments and their impact on the structure of the Cs_0.17_FA_0.83_PbI_3_/copper interface. (a) Reactions during and after evaporation of copper. (b) Reactions during initial exposure to air. (c) Long-term reactions leading to the formation of [Pb_4_(OH)_4_][Cu_2_I_6_] (siidraite).

The degradation of copper and perovskite appears to begin during evaporation, resulting in the formation of metallic lead, copper iodide, and a new nitrogen species, shown schematically in Figure 4a. The evaporation of copper on perovskite and lead iodide surfaces appears to result in the following reaction:
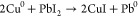
which is endothermic (Δ*H* = +40 kJ/mol) and not expected to be spontaneous (Table S1).^49^ Furthermore, the reaction occurs only during evaporation and not during subsequent measurements in a vacuum, even though both reactants can still be detected. This suggests that this initial endothermic reaction is driven by deposition (desublimation) of monatomic copper on the surface (337.2 kJ/mol).^50^ The degradation of the copper is also driven by a reaction with the organic cation (FA^+^). The latter is indicated by the formation of a new, lower binding energy nitrogen species after evaporation of copper.

However, the most significant degradation occurs during exposure to air (schematically shown in Figure 4b). When the samples are exposed to air, the oxidation of Pb^0^ and the formation of Pb–O containing compounds is observed, although exactly which compounds form is not known. Furthermore, degradation continues even after the Pb^0^ formed during evaporation has been oxidized, as we are able to observe the formation of additional Pb–O compounds and the formation of CuI. The reaction in air, expected to be exothermic (Table S1), results in the degradation of the surface perovskite and likely also results in the bulk degradation of the perovskite as we will show below. After being exposed to air, significantly more of the new nitrogen species forms and it also appears at higher binding energy, indicating an effect of either oxidation or moisture. Similar behavior is also observed with FAI, where the degradation of copper appears to be significantly more rapid than that with PbI_2_. Furthermore, the reaction with the cation is limited to FA^+^ as no new nitrogen species is formed when copper is evaporated on MAPbI_3_.

The ex situ measurements of FAI show that the new nitrogen initially forms with a nitrogen to iodide ratio of 2:1, although this ratio changes over time, suggesting that nitrogen and iodide are not directly bound to each other (as in, e.g., CuI). However, the compound appears to have a constant Cu(I)/N ratio between 1 and 2 and does not contain any Cu(II). Additionally, the compound is amorphous or weakly diffracting, as will be shown below. However, this is not sufficient information for any confident assignment of the new nitrogen species. We have previously observed the formation of similar nitrogen compounds during atomic layer deposition of SnOx, although in this case, the process appears to be different.^51^ The new species could be deprotonated FA (CHNH_2_NH); however, the shift from deprotonation is expected to be about 2 eV, compared to a shift of around 3 eV observed in our measurements.^52,53^ This is further complicated by the apparent lack of studies of the interaction of formamidine or formamidinium with metals, in general, and copper, specifically. However, we recognize two possibilities: the formation of copper–nitrogen complexes (including metal organic frameworks) or the transformation of FA^+^ into new compounds (including polymerization of FA^+^). These options are also not mutually exclusive, as the transformation of FA^+^ might be followed by the formation of copper–nitrogen complexes.

The formation of copper–nitrogen complexes from a reaction between the evaporated copper and FA^+^ cation in the perovskite would be expected as copper is known to readily form relatively stable complexes with nitrogen compounds.^54^ One possibility is the formation copper(I)halide nitrogen complexes, many of which result in the formation of a metal–organic framework of which there is a large variation.^55,56^ The closest structures we were able to find in the literature are bis(guanidinium) copper(I) iodide^57^ as well as some copper amidinates.^58^ Furthermore, copper(I) halide complexes containing N–H groups can be sensitive to oxygen, which could explain the decrease in the I/N ratio and why we observe a shift in the binding energy after exposure to air.^56^ However, most (but not all) copper–nitrogen complexes in the literature have a binding energy in the region of 399–401 eV, that is, at slightly higher binding energy than observed here.^59−61^ Furthermore, it is not certain that the formation of copper–nitrogen bonds would result in a shift to lower binding energies.^62^

The presence of copper compounds (CuI and Cu_2_O) has been shown to be catalytic for many organic compounds, and as a result, we may have transformation of FA^+^ to new compounds. Among others, CuI nanoparticles have been shown to catalyze reactions resulting in new C–N bonds,^63^ and calculations show that CuI increases the reactivity of *N*,*N*′-diisopropylcarbodiimide,^64^ which has a structure similar to that of FA^+^. In addition, metal oxides, expected to form due to exposure to air, catalyze the degradation of the FA^+^ cation, especially at higher temperatures. Depending on the metal oxide and temperature, this results in the formation of sym-triazine (HCN)_3_, ammonium iodide (NH_4_I), ammonia (NH_3_), water (H_2_O), and hydrogen iodide (HI).^65^ However, these reactions have also been shown to occur at higher temperature without metal oxide catalysts.^66^ Compounds such as sym-triazine should be detected at binding energies significantly higher than what we observe.^62^ Furthermore, the presence of catalysts on the surface could potentially result in the polymerization of FA^+^. Many nitrogen-containing polymers readily form bonds with copper, which can result in binding energies similar to what we observe.^67,68^ To summarize, there is a wide range of candidates for the new nitrogen species, especially given the reactivity of nitrogen compounds with copper and catalytic properties of copper compounds. This is beyond the present investigation, and further studies using more specialized techniques might be needed to identify this new nitrogen compound.

### 3.7 Bulk Degradation

**Figure 5:**
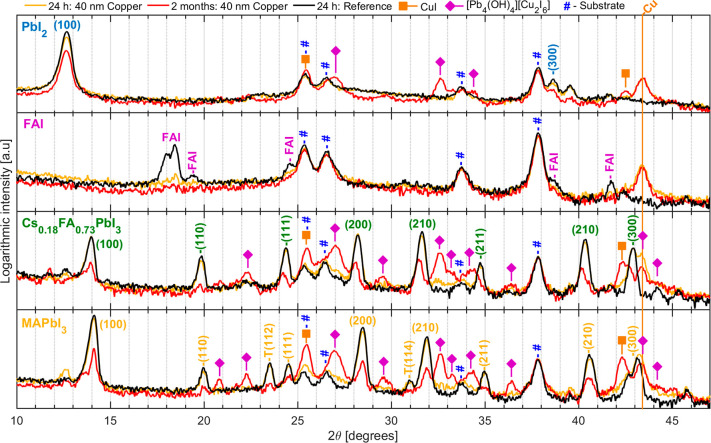
X-ray diffractograms (counts on log scale) of thin film samples with and without copper, measured using Cu Kα with a θ of 2°. The PbI_2_ and perovskites are assigned to the cubic phase, with the exception two peaks from the tetragonal phase which are marked with T. Diffractograms with linear scale are shown in Figure S10.

While the above clearly shows that the perovskite degrades at the interface with copper, it is also interesting to understand the long-term impact of the reaction on the bulk of the perovskite layers. To investigate this, we studied our ex situ samples of MAPbI_3_ and Cs_0.17_FA_0.83_PbI_3_ as well as the precursors PbI_2_ and FAI with 40 nm evaporated copper using XRD analysis within 24 h and 2 months after evaporation. Half of each sample had been masked during evaporation, enabling us to compare the changes in the perovskite with copper to a part of the same perovskite sample without copper. The samples were stored in air, ensuring access to both atmospheric oxygen and humidity, but enclosed in separate containers to prevent cross-contamination between the samples. Figure 5 shows the diffractograms of the PbI_2_, FAI, Cs_0.17_FA_0.83_PbI_3_, and MAPbI_3_ samples on areas with and without 40 nm of copper. The measurements of the regions without copper 2 months after evaporation are not shown within this figure, as no significant changes were observed in these regions (Figure S9).

In areas where no copper was evaporated, we observe the diffraction peaks of the substrate (TiO_2_ and FTO), indicated with #, and of the compounds PbI_2_ (PDF 04-009-6354), FAI,^65^ Cs_0.17_FA_0.83_PbI_3_ (PDF 00-069-0999), and MAPbI_3_ (PDF 01-085-5508). In areas where copper was evaporated, we observe the appearance of diffraction peaks from metallic copper, as indicated with Cu (PDF 00-004-0836), as well as the peaks of the substrate. There is very little change in the diffraction peaks of the PbI_2_, Cs_0.17_FA_0.83_PbI_3_, and MAPbI_3_ immediately after evaporation, indicating no significant short-term changes to the bulk crystalline properties in response to the deposition of copper. However, we find that the FAI diffraction peaks have almost completely disappeared already within 24 h of evaporation. We can rule out the attenuation of the X-rays by the deposited copper because there is very little change in the peaks of substrate (FTO and TiO_2_). Instead, a more likely explanation is the formation of an amorphous compound in the reaction between copper and FAI.

After 2 months, there is a significant decrease in the diffraction peaks originating from the PbI_2_, Cs_0.17_FA_0.83_PbI_3_, and MAPbI_3_ materials, indicating that the presence of the evaporated copper induces significant long-term bulk degradation. Notable is the absence of any diffraction peaks of degradation products such as PbI_2_ and PbO. However, there is a number of new diffraction peaks after 2 months that are not detected in the region without copper. Some of the peaks can be assigned to CuI (PDF 00-006-0246), marked with orange square, which is expected from the reaction of copper with an iodide-rich material. Interestingly, however, the remaining new peaks can all be attributed to siidraite (PDF 04-022-2017), a mineral which has the chemical structure [Pb_4_(OH)_4_][Cu_2_I_6_] and is marked with purple diamonds in the diffractogram.

Siidraite consists of a checkerboard pattern of [Pb_4_(OH)_4_]^4+^ cubane groups and [Cu_2_I_6_]^4–^ dimers where two iodides are shared by the copper atoms. It occurs naturally as rare secondary phase together with cuprite (Cu_2_O), anglesite (PbSO_4_), marchite (CuI), and galena (PbS).^69,70^ Additionally, two groups have synthesized the material in the lab. Xue et al. fabricated siidraite by ionothermal synthesis by heating a mixture of PbI_2_, CuI, H_2_O, and 1-ethyl-3-methyl imidazolium iodide in an autoclave at 180 °C for 4 days.^71^ Pan et al. fabricated the material by heating a 2:1 mixture CuI and Pb(NO_3_)_2_ mixture in a solution of *N*,*N*′-dimethylformamide/water at 90 °C for 2 days.^72^ Once fabricated, the material shows high stability, being able to withstand 250 °C in nitrogen atmosphere^71^ and being submerged in water for several days, even in boiling water for 4 h.^72^

We have already shown the formation of CuI and Pb–O compounds during evaporation of copper and exposure to air. It is likely that the interactions between these compounds are an important step for the bulk degradation of the perovskite, shown in Figure 4c. However, the exact reaction is not clear as all known synthesis methods use an aqueous solution. This is because one of the main components, the Pb_4_(OH)_4_^4+^ clusters, forms in aqueous solution at pH between 6 and 11.^73^ It is possible that there is some humidity present on the sample which could result in the formation of these clusters. However, the FA^+^/MA^+^ in the perovskite could also function as a source of H^+^ for the formation of Pb_4_(OH)_4_ from Pb–O compounds.

## 4 Conclusion

We evaporated copper on the surface of a perovskite with the composition Cs_0.17_FA_0.83_PbI_3_, and using in situ photoelectron spectroscopy with support from XRD, we were able to study the rich and complex chemistry that occurred in the interface between the two materials. This chemistry is summarized in the illustration shown in Figure 4. This figure illustrates the short-term degradation of the perovskite at the interface, first initiated during the evaporation of the copper and then completed by the exposure to oxygen and moisture from the air. This is followed by long-term degradation of the perovskite, leading to a significant degradation of the perovskite bulk and the formation of CuI and [Pb_4_(OH)_4_][Cu_2_I_6_]. The degradation of the perovskite appears to follow two different pathways, one involving reactions with the lead halide cage and one involving reactions with the organic FA^+^ cation.

The reaction between copper and the lead halide cage starts during the evaporation, where it results in an initial formation of CuI and Pb^0^. Thereafter, the interface remains relatively inert until it is exposed to atmosphere. However, when the sample is exposed to air, we observe the degradation of the remaining perovskite at the surface and the oxidation of the metallic lead, formed at the surface during evaporation, resulting in the formation of Pb–O compounds. This degradation continues as long as the sample is exposed to air, leading to the formation of more CuI and Pb–O compounds and eventually [Pb_4_(OH)_4_][Cu_2_I_6_], also known as siidraite. If allowed to continue, this will result in bulk degradation of the perovskite (or PbI_2_). The reaction between the organic cation and copper occurs during evaporation, indicated by the appearance of new nitrogen species in both Cs_0.17_FA_0.83_PbI_3_ and FAI. Upon exposure to air, there is a significant increase in intensity and shift to higher binding energies of the new nitrogen signal, indicating significant formation of this new species but also a change in the chemical character, possible due to a reaction with the oxygen or moisture in the air. Furthermore, it appears that the formation of this new nitrogen species continues as the sample is exposed to air. Although we are unable to identify the new nitrogen species, we have some important clues which might help guide further research. The new species appears to be amorphous and appears with Cu(I) in a ratio slightly above 1 Cu(I)/N. Perhaps most significant is that no new nitrogen species are detected in MAPbI_3_, indicating that this type of reaction does not occur with MA^+^.

These results also have some implication on the viability of copper as an electrode for lead halide perovskite solar cells and can guide the design of copper-based electrodes. The initial formation of metallic lead during evaporation requires a bare perovskite surface and should therefore be blocked by the presence of a selective contact. The presence of an interlayer could help prevent the formation of Pb–O compounds and consequently the formation of [Pb_4_(OH)_4_][Cu_2_I_6_] as this reaction is likely to require direct contact between the perovskite and CuI. With a continuous interlayer, different reactions are potentially dominant, which could explain the discrepancies between the rapid degradation we observe and the reported stability of Cu electrodes in the literature.^11,13−16^ However, this might not be the case for the FA^+^ or I^–^ which, depending on the selective contact, could diffuse to the copper electrode and induce degradation. The reactivity of FA^+^ with copper might explain the degradation observed in a perovskite with FA^+^ but not in one with only MA^+^ when used with C_60_ as a selective contact.^16^ The diffusion of copper ions into the perovskite, similar to what is observed with Au^7^ and Ag,^36^ might also be a problem. However, effective encapsulation of the device should block both surface and bulk degradation as this only occurs when the sample is exposed to air. This is supported by other studies showing that copper is stable in contact with perovskite given that exposure to air/oxygen is avoided.^11,12^ To conclude, copper has the potential to be a good electrode material capable of replacing Au in real PV applications but does not live up to the idealized performance speculated on in the Introduction. Indicated from the results reported here, copper in direct contact with the perovskite, especially the FA-containing lead halide perovskites used in most of the best research cells, does not lead to benign self-passivation as has been speculated but instead results in perovskite degradation and the formation of new compounds. Careful design of the interlayers is therefore required to prevent the direct contact between the copper electrodes and the perovskite to ensure long-term stability.

## Supporting Information Available

Table of enthalpies for the potential reactions between the PbI_2_ and copper; X-ray photoelectron spectroscopy spectra of the in situ perovskite samples (effect of sputtering, spectral evolution, long-term air exposure, Cu 2p core level and nonsputtered sample), X-ray photoelectron spectroscopy spectra of the ex situ samples (core levels of Cs_0.17_FA_0.83_PbI_3_ and MAPI_3_ and Fermi edge of FAI); X-ray diffraction (logarithmic scale) of the clean thin films within 24 h and 2 months; X-ray diffraction (linear scale) of the thin films with and without copper within 24 h and 2 months (PDF)
