# Supplementary material for: The Complex Degradation Mechanism of Copper Electrodes on Lead Halide Perovskites
Source: ACS Mater Au. 2022 Feb 2;2(3):301–12. doi: 10.1021/acsmaterialsau.1c00038 (PMC9100662; doi:10.1021/acsmaterialsau.1c00038)
Supplement: Supplementary file 1 — mg1c00038_si_001.pdf [file mg1c00038_si_001.pdf]

Supporting Information

The complex degradation mechanism of copper electrodes on lead halide perovskites

Sebastian Svanström,[1] Alberto García-Fernández,[2] T. Jesper Jacobsson,[3] Ieva Bidermane,[4] Torsten Leitner,[4] Tamara Sloboda,[2] Gabriel J. Man,[1] Gerrit Boschloo,[5] Erik Johansson,[5] Håkan Rensmo[*,1] Ute B. Cappel[*,2]

1: Condensed Matter Physics of Energy Materials, Division of X-ray Photon Science, Department of Physics and Astronomy, Uppsala University, Box 516, SE-751 20, Uppsala, Sweden

2: Division of Applied Physical Chemistry, Department of Chemistry, KTH - Royal Institute of Technology, SE-100 44 Stockholm, Sweden

3: Young Investigator Group Hybrid Materials Formation and Scaling, Helmholtz-Zentrum Berlin für Materialen und Energie GmbH, Albert-Einstein Straße 15, 12489 Berlin, Germany

4: Uppsala-Berlin Joint Laboratory on Next Generation Photoelectron Spectroscopy, Albert-Einstein-Str. 15, 12489 Berlin, Germany

5: Dept. of Chemistry, Uppsala University, Box 538, 75121 Uppsala, Sweden

* cappel@kth.se

*hakan.rensmo@physics.uu.se

Energy calculations of reactions

Table S1: Change in enthalpy of selected reactions under standard conditions.[1]

Description:	Reaction:	Enthalpy [kJ/mol]	Gibbs free energy [kJ/mol]
Formation of Cu_2O	$2Cu + \frac{1}{2}O_2 \rightarrow Cu_2O$	-168.6	-146.0
Formation of CuO	$Cu + \frac{1}{2}O_2 \rightarrow CuO$	-157.3	-129.7
Formation of CuI	$Cu + \frac{1}{2}I_2 \rightarrow CuI$	-67.8	-69.5
Formation of PbI_2	$Pb + I_2 \rightarrow PbI_2$	-175.5	-173.6
Formation of PbO	$Pb + \frac{1}{2}O_2 \rightarrow PbO$	-219.0	-188.9
Reaction between Cu and PbI_2	$2Cu + PbI_2 \rightarrow 2CuI + Pb$	+39.9	+34.6
Reaction between Cu and PbI_2 with O_2	$2Cu + PbI_2 + \frac{1}{2}O_2 \rightarrow 2CuI + PbO$	-179.1	-154.3

In-situ samples

Figure S1 shows the Cs4d, I4d, Pb5d, O2s, N1s, C1s and $Pb4f_{7/2}$ core levels before and after sputtering, normalised to the Pb^{2+} component of $Pb4f_{7/2}$. After sputtering there is a significant decrease in the C1s core level intensity associated with adventitious carbon and in the broad O2s core level intensity due to surface oxides and oxygen containing compounds being removed. There is also the appearance of a N1s core level at 400.8 eV associated with the FA^+ which indicates that the surface now consists of perovskite. There is also an increase in the I4d signal, indicating that the surface contains more I in relation to Pb^{2+} after sputtering.

Furthermore, we observe the formation of a small amount (about 10 % of total Pb) of metallic lead, indicating that sputtering causes some damage to the perovskite in addition to cleaning the surface. However, the sputtering appears to remove the majority of surface contaminants and the annealed sample shows core levels at position expected for a perovskite. The sample was annealed by heating to 90 ^{o}C in vacuum and allowed to cool before copper was thermally evaporated. There was little to no change in the spectra during or after annealing.

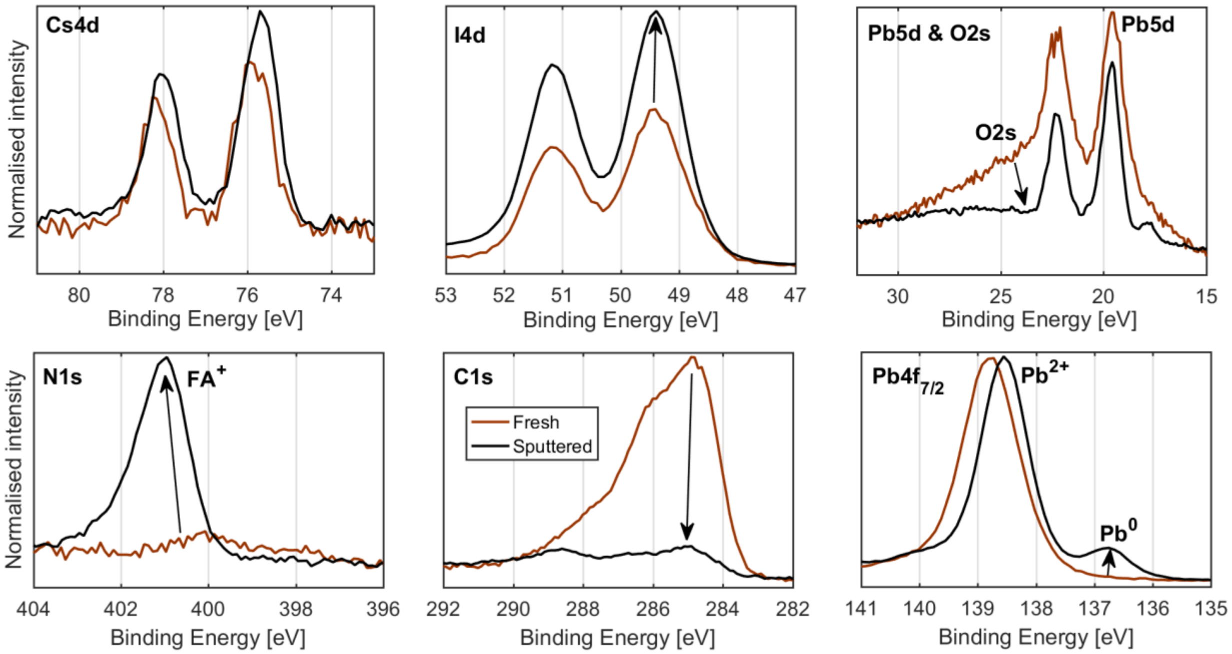

Figure S1: Cs4d, I4d, Pb5d, O2s, N1s, C1s and $Pb4f_{7/2}$ core levels of $Cs_{0.17}FA_{0.83}PbI_3$ normalised to Pb^{2+} peak height from Pb4f recorded with a photon energy of 535eV. The N1s and C1s core levels were measured using ArTOF2-EW and the remaining core levels were measured using ArTOF-10k. The binding energy was calibrated by putting $I4d_{5/2}$ at 49.4 eV.

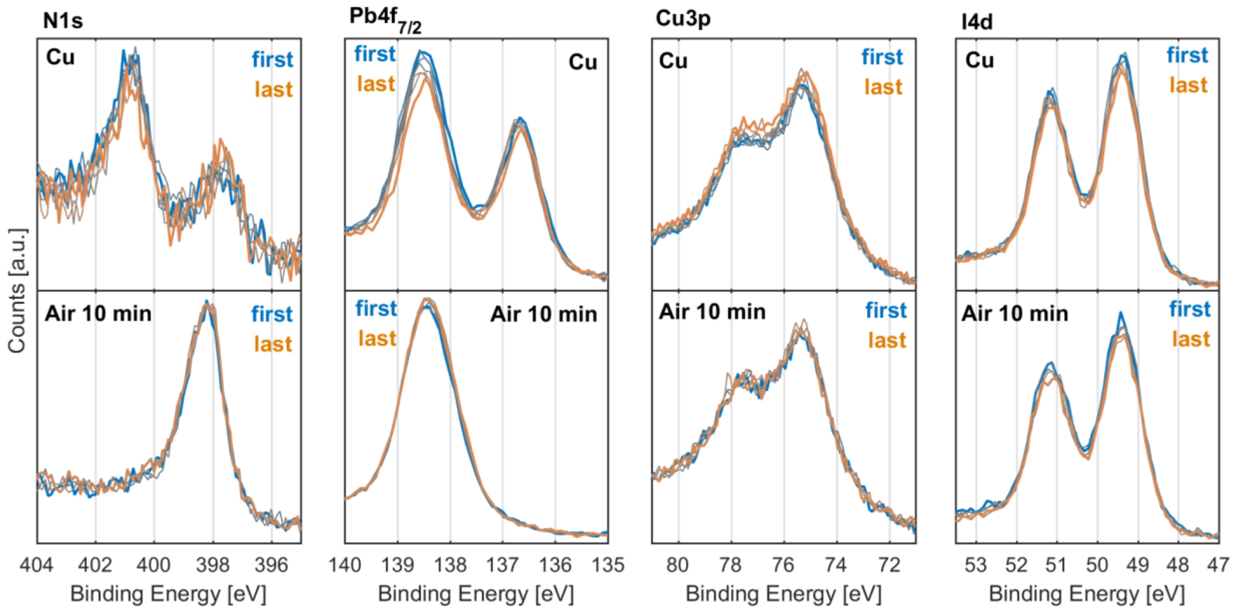

Figure S2: The spectral evolution of the N1, Pb4f$_{7/2}$, Cu3p and I4d signals of the $Cs_{0.17}FA_{0.83}PbI_3$ sample after evaporation of copper (top) and exposure to air for 10 minutes (bottom). The total measurement time is 90 min after copper evaporation and 60 min after exposure to air for 10 min. No significant spectral changes are observed, confirming that the sample surfaces did not change during measurement.

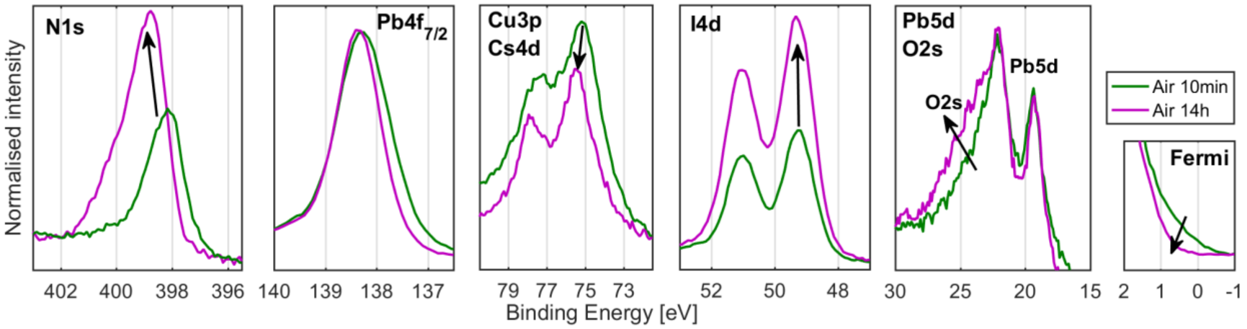

Figure S3: The N1, Pb4f$_{7/2}$, Cu3p and I4d core levels of the $Cs_{0.17}FA_{0.83}PbI_3$ sample after exposure to air for 10 minutes and 14 hours recorded with a photon energy of 535 eV. Intensity normalised against Pb4f$_{7/2}$ peak height and energy calibrated against I4d at 49.4 eV. We observe the disappearance of the Fermi edge, a decrease in the Cu3p signal and an increase in I4d signal, relative to Pb^{2+}. This suggests the formation of more CuI at the surface as there is more I^- not bound to Pb^{2+} together with the oxidation of metallic copper at the surface. At the same time there is an increase in the O2s signal, suggesting the formation of more Pb-O or Cu-O compounds. Finally, we also observe an increase in intensity, and a shift in towards higher binding energies, of the new nitrogen to higher binding energies.

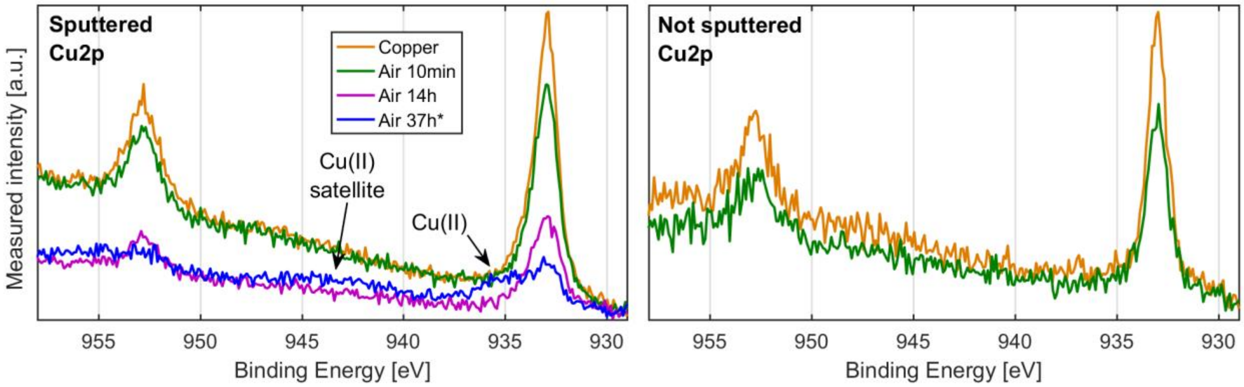

Figure S4: The Cu2p core level of the sputtered and not sputtered sample measured using the ArTOF-10k with a photon energy of 1060 eV. Binding energy is calibrated by putting $Cu2p_{3/2}$ at 933 eV. *from a separate sample. A Cu(II) signal is first observed in the measurement 37 hours after evaporation.

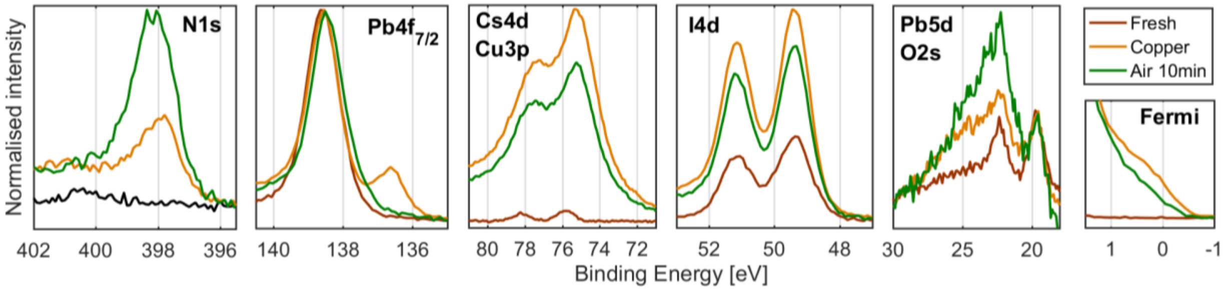

Figure S5: N1s, Cu3p, Cs4d, I4d, Pb5d, O2s, Pb4f core levels and Fermi level normalised to Pb^{+2} component from Pb4f recorded for a $Cs_{0.17}FA_{0.83}PbI_3$ sample, which was not sputtered, with a photon energy of 535 eV. The evaporation and air exposure procedure were identical to that of the sputtered sample. The N1s core levels were measured using ArTOF-10k, with the remaining core levels using ArTOF2-EW. The measurements were carried out with a photon energy of 535 eV and the binding energy was calibrated by putting I4d at 49.4 eV. There are a number of differences compared to the sputtered sample in the reaction after evaporation: A smaller amount of Pb^0 is formed after copper evaporation (about 8 % of total Pb); the new nitrogen species is at a higher binding energy after copper evaporation, but identical to the position after air exposure; a smaller decrease in the intensity of Fermi level after exposure to air than for the sputtered sample.

Ex-situ samples

X-ray photoelectron spectroscopy

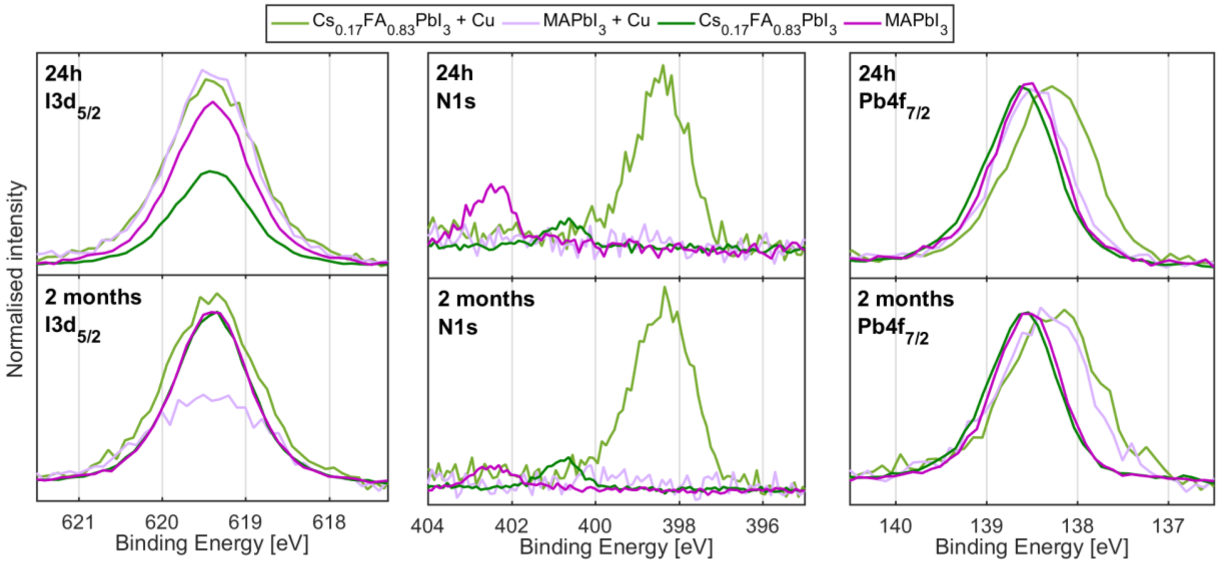

Figure S6: The $I3d_{5/2}$, N1s and $Pb4f_{7/2}$ core level spectra of the $Cs_{0.17}FA_{0.83}PbI_3$ and $MAPbI_3$ samples 24 hours and 2 months after evaporation, with and without 40 nm of copper deposited recorded with a photon energy of 1486.6 eV. Intensity normalised against $Pb4f_{7/2}$ and energy calibrated against $I3d_{5/2}$ at 619.4 eV.

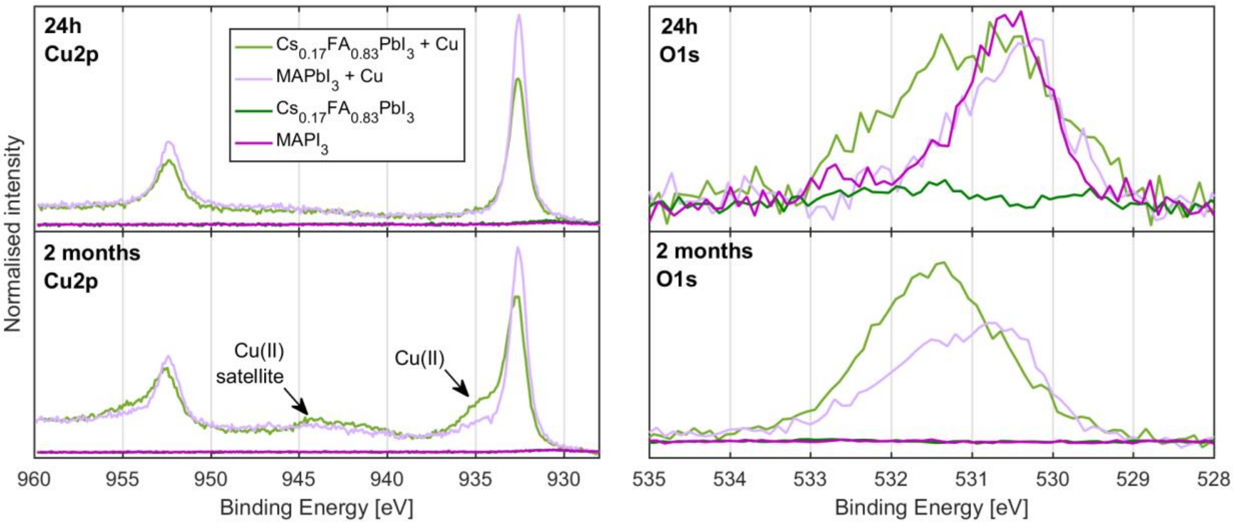

Figure S7: The Cu2p and O1s core level spectra of the $Cs_{0.17}FA_{0.83}PbI_3$ and $MAPbI_3$ samples 24 hours and 2 months after evaporation, with and without 40 nm of copper deposited recorded with a photon energy of 1486.6 eV. Intensity normalised against $Pb4f_{7/2}$ and energy calibrated against $I3d_{5/2}$ at 619.4 eV.

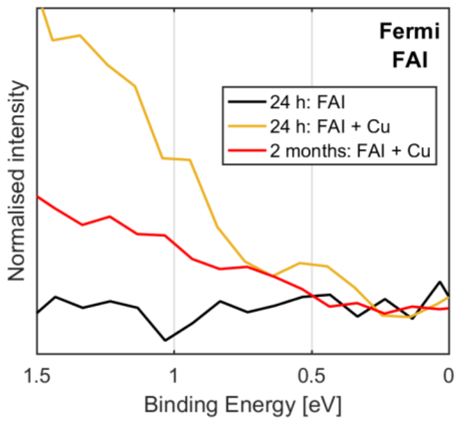

Figure S8: The Fermi edge of the FAI sample 24 hours and 2 months after evaporation, with and without 40 nm of copper deposited. Intensity normalised against total N1s signal and energy calibrated against $I3d_{5/2}$ at 619.4 eV recorded with a photon energy of 1486.6 eV.

X-ray diffraction

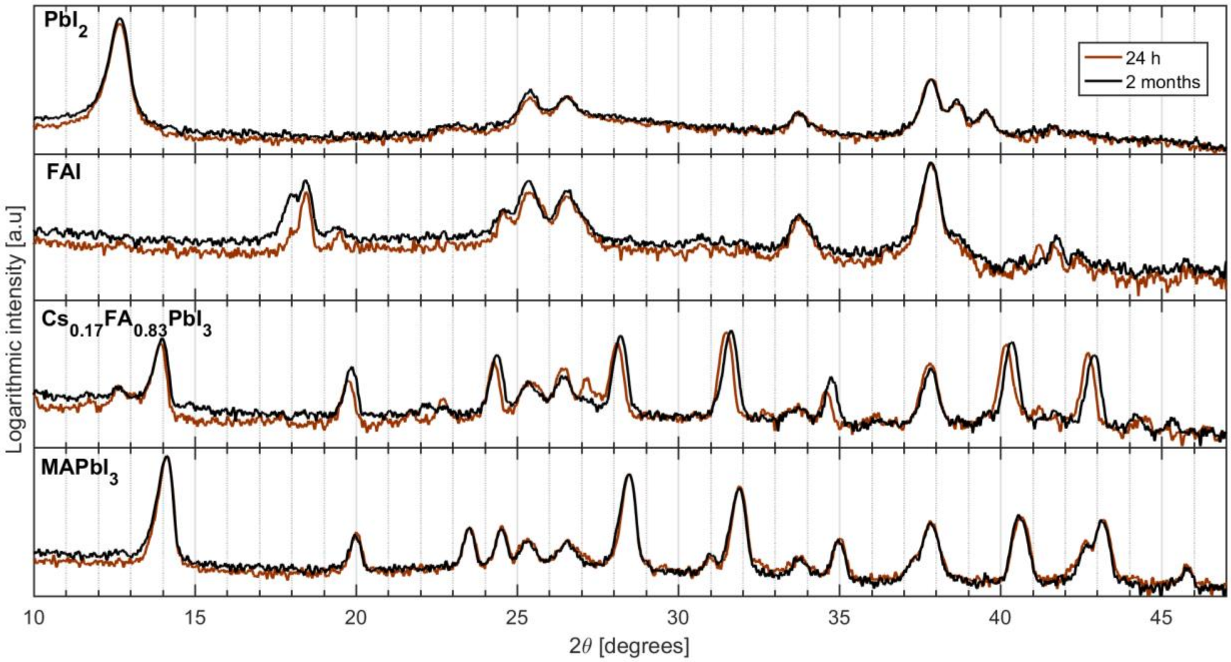

Figure S9: X-ray diffractogram (counts on log scale) of the reference samples 24 hours and 2 months after copper evaporation measured using CuKα with a θ of 2°.

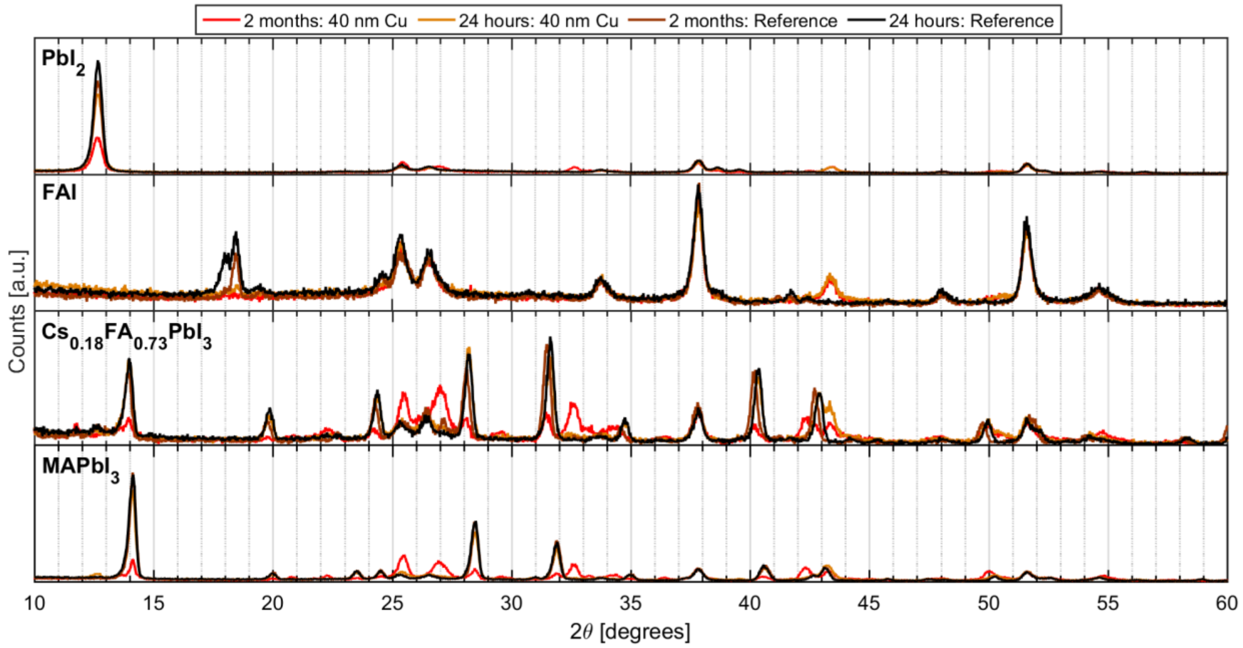

Figure S10: X-ray diffractogram of the compounds with and without copper, 24 hours and 2 months after copper evaporation measured using CuKα with a θ of 2°.

References

(1) Daubert, T. E.; Danner, R. P. Physical and Thermodynamic Properties of Pure Compounds: Data Compilation. *Hemisphere N. Y.* **1989**, *2001*.
